# Linked Patient-Reported Outcomes Data From Patients With Multiple Sclerosis Recruited on an Open Internet Platform to Health Care Claims Databases Identifies a Representative Population for Real-Life Data Analysis in Multiple Sclerosis

**DOI:** 10.2196/jmir.5805

**Published:** 2016-09-22

**Authors:** Valery Risson, Bhaskar Ghodge, Ian C Bonzani, Jonathan R Korn, Jennie Medin, Tanmay Saraykar, Souvik Sengupta, Deepanshu Saini, Melvin Olson

**Affiliations:** ^1^ Novartis Pharma AG Basel Switzerland; ^2^ IMS Health Basel Switzerland; ^3^ IMS Health London United Kingdom; ^4^ IMS Health Burlington, MA United States; ^5^ IMS Health Parsippany, NJ United States; ^6^ IMS Health Gurgaon, Delhi India

**Keywords:** Internet, multiple sclerosis, outcomes assessment, linkage analysis

## Abstract

**Background:**

An enormous amount of information relevant to public health is being generated directly by online communities.

**Objective:**

To explore the feasibility of creating a dataset that links patient-reported outcomes data, from a Web-based survey of US patients with multiple sclerosis (MS) recruited on open Internet platforms, to health care utilization information from health care claims databases. The dataset was generated by linkage analysis to a broader MS population in the United States using both pharmacy and medical claims data sources.

**Methods:**

US Facebook users with an interest in MS were alerted to a patient-reported survey by targeted advertisements. Eligibility criteria were diagnosis of MS by a specialist (primary progressive, relapsing-remitting, or secondary progressive), ≥12-month history of disease, age 18-65 years, and commercial health insurance. Participants completed a questionnaire including data on demographic and disease characteristics, current and earlier therapies, relapses, disability, health-related quality of life, and employment status and productivity. A unique anonymous profile was generated for each survey respondent. Each anonymous profile was linked to a number of medical and pharmacy claims datasets in the United States. Linkage rates were assessed and survey respondents’ representativeness was evaluated based on differences in the distribution of characteristics between the linked survey population and the general MS population in the claims databases.

**Results:**

The advertisement was placed on 1,063,973 Facebook users’ pages generating 68,674 clicks, 3719 survey attempts, and 651 successfully completed surveys, of which 440 could be linked to any of the claims databases for 2014 or 2015 (67.6% linkage rate). Overall, no significant differences were found between patients who were linked and not linked for educational status, ethnicity, current or prior disease-modifying therapy (DMT) treatment, or presence of a relapse in the last 12 months. The frequencies of the most common MS symptoms did not differ significantly between linked patients and the general MS population in the databases. Linked patients were slightly younger and less likely to be men than those who were not linkable.

**Conclusions:**

Linking patient-reported outcomes data, from a Web-based survey of US patients with MS recruited on open Internet platforms, to health care utilization information from claims databases may enable rapid generation of a large population of representative patients with MS suitable for outcomes analysis.

## Introduction

The Internet and social media are driving a revolution in communication and information sharing, with a fundamental impact on health care. Patients’ voices have become more influential through the exchange of information in the form of conversations, blogs, tweets, and other postings on social media. This development is changing the power balance in decisions regarding health care, requiring traditional stakeholders to recognize patients’ perspectives in the provision and evaluation of treatments [1-3].

An enormous amount of information relevant to public health is being generated directly by online communities [4,5]. Epidemiology [6,7], pharmacovigilance [8,9], identification of malpractice [10], and the support of health behavior changes [11] are only a few examples of areas where informal data have been successfully applied. Moreover, the Internet and particularly social networks represent a large number of individuals with shared interests, nationalities, or characteristics that can be reached by relatively modest financial or human resources.

We have previously reported on the feasibility of applying social media listening (defined as the mining and analysis of information gathered from social media) to retrospective analyses in outcomes research, specifically the use of patient-reported reasons for switching between different treatment modalities for multiple sclerosis (MS) [12]. The ability to include patient-reported information to enhance prospective analyses of sources such as claims databases would appear to have great promise in outcomes research. We present here an approach to create a dataset that contains both patient outcomes data (from a Web-based survey of US patients with MS recruited on an open Internet platform) and health care utilization information from claims databases. A linkage analysis has recently been performed on data from dedicated patient platforms and invited patients [13]. We hypothesized that linking patient data from the social media survey with those from the claims databases could identify a representative population that can be used for real-life data analysis in MS. The initial analysis focused on verifying the method by demonstrating that the characteristics of the linked population recruited on the open Internet platform are representative of the MS population in the United States.

## Methods

### Study Aim and Design

The primary aim of this pilot study was to explore the feasibility of creating a dataset that links patient-reported outcomes data, from a Web-based survey of US patients with MS recruited on open Internet platforms, to health care utilization information from health care claims databases. The representativeness of the linked populations was validated by a comparison with the characteristics of known MS populations in the United States.

This study was designed, implemented, and reported in accordance with the Guidelines for Good Pharmacoepidemiology Practices of the International Society for Pharmacoepidemiology [14], the Strengthening the Reporting of Observational Studies in Epidemiology (STROBE) guidelines [15], and the ethical principles laid down in the Declaration of Helsinki [16]. The secondary data source used for the analysis meets all of the US Health Insurance Portability and Accountability Act (HIPAA) compliance standards, ensuring patient anonymity. As such, approval from an institutional review board was not necessary.

The defined target population was a broad, US-based, commercially insured population with MS diagnosed and treated by a specialist. All participants took part in the survey entirely of their own volition, and complete information regarding how the data would be used was provided before patients agreed to take part. Full anonymity was guaranteed at all points of the process of running the survey and performing the linkage and subsequent analysis.

### Recruitment and Survey

**Figure 1:**
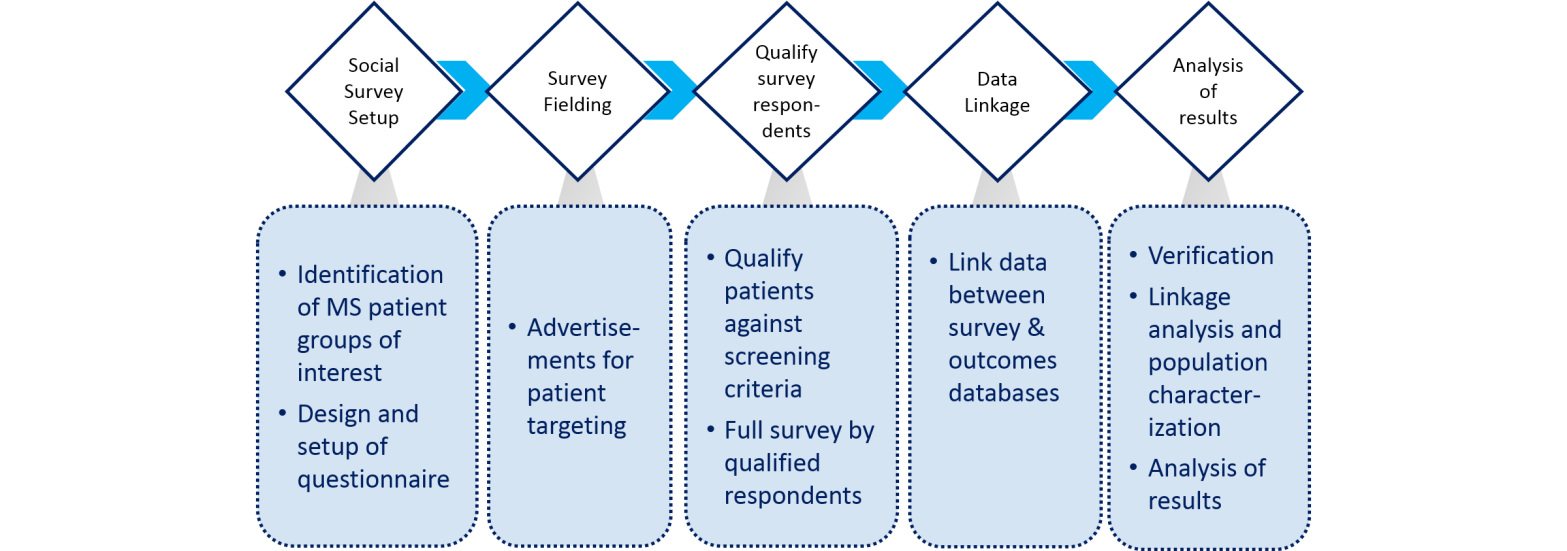
Survey and linkage analysis process. MS: multiple sclerosis.

The survey process is shown schematically in Figure 1. US Facebook users with an interest in MS were alerted to a patient-reported survey by targeted advertisements. The identification of users with a high interest in MS for the placement of advertisements was performed by Facebook as a commercial service and was beyond the control of the researchers. Users clicking on the survey advertisement were provided a disclaimer on the study and how data would be used, followed by options to decline or consent to proceed to the survey. Users were anonymous until the time at which they consented to taking the survey and passed the screening criteria. No identifiable information was collected from users who declined to take the survey. Users who agreed to participate were redirected away from the Facebook domain to the survey, which was hosted on a secure third-party site accessed using an https (hypertext transfer protocol, secure) protocol. Neither the advertisements nor the survey was branded by any commercial entity.

Before completing the survey, patients were screened for eligibility. Screening questions are presented in Textbox 1. The predefined criteria were a diagnosis of MS by a specialist; ≥12 months of history of diagnosed disease before taking the survey; 18-65 years of age; and commercial health insurance, both current and in the ≥12 months before study entry. The disease could be primary progressive, relapsing-remitting, or secondary progressive MS.

**Screening questions.**

Screener questions

In which country do you currently live?

(*In the US* / Outside of the US)

Are you currently between the ages of 18 and 65 years of age?

(*Yes* / No)

Have you been diagnosed with Multiple Sclerosis by a specialist?

(*Yes* / No / Not Sure)

How long have you had diagnosed Multiple Sclerosis?

(< 1 year, *> 1 year*, *> 5 years*, *> 10 years*)

What type of Multiple Sclerosis do you have?

(*Relapsing-remitting Multiple Sclerosis (RRMS)*, *Primary Progressive Multiple Sclerosis (PPMS)*, *Secondary Progressive Multiple Sclerosis (SPMS)*, I don’t know)

Do you have health insurance through a commercial health plan?

(*Yes*, No—I currently do not have health insurance, No—I am on a Medicare or Medicaid health plan)

How long have you been with your current health insurance provider?

(< 1 year, *> 1 year*, *> 5 years*, *> 10 years*)

If the defined criteria (italics) are not met, the patient will be excluded from the survey.

The survey was designed to provide information on demographics and disease characteristics, current and earlier therapy use, relapses, disease severity and disability, health-related quality of life (on the EQ-5D-3L and EQ-VAS scales), and employment status and productivity. The survey is included in Multimedia Appendix 1. Survey participants were informed about data handling, anonymity, and the right to revoke consent in a disclaimer, provided in Multimedia Appendix 2.

All data were hosted in a secure data enclave using network firewalls. Access was provided only to named users who had successfully completed training on the handling of health information and signed data nondisclosure agreements. Before the linkage testing, all data collected from the Web-based survey were deidentified by a trusted third party (Management Science Associates Inc, Pittsburgh, PA), using patient deidentification software, encrypting patients’ protected health information data elements in accordance with the Expert Determination De-Identification methodology of the HIPAA Privacy Rule law. The linkage to claims data and subsequent analyses were performed on the deidentified survey population.

### Linkage Analysis

**Figure 2:**
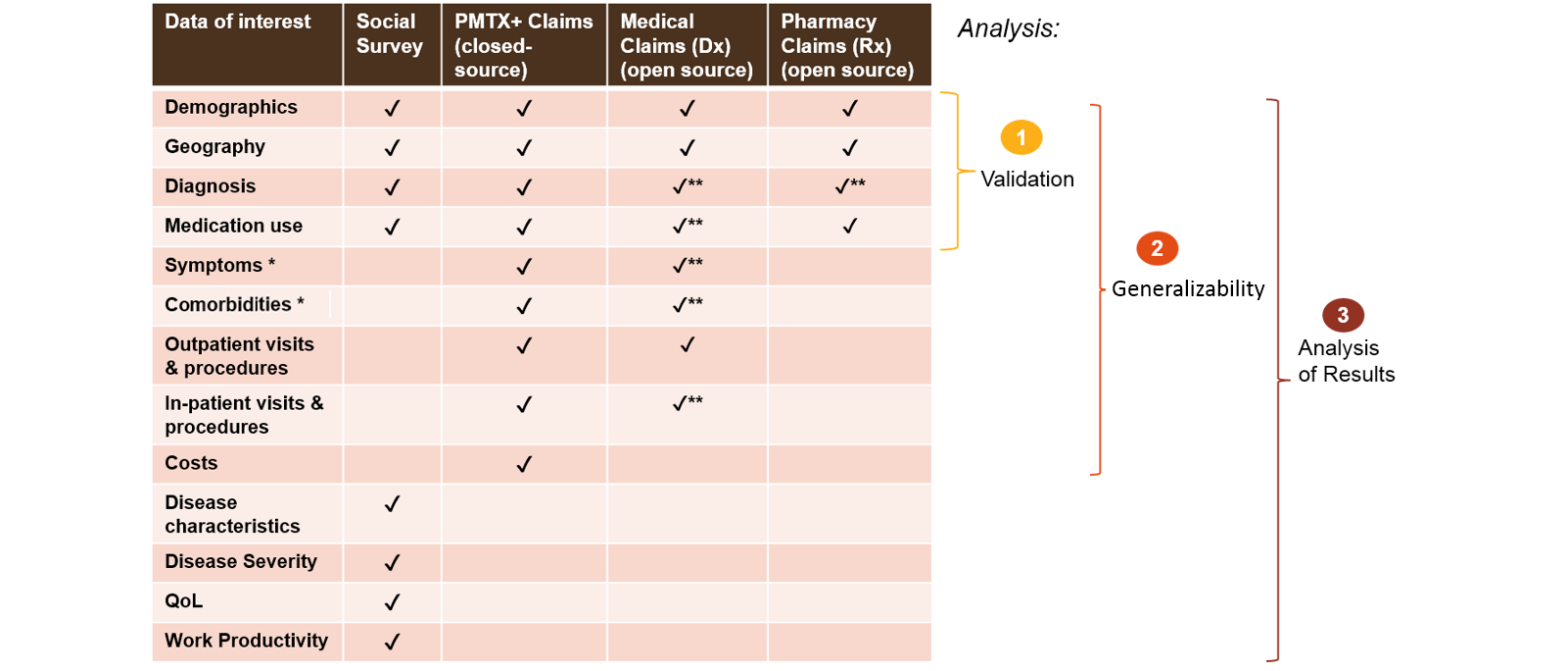
Overview of the data available in the different sources included in the linkage analysis. The cohorts identified in the medical claims (Dx) and prescription claims (Rx) databases were merged for the linkage analysis. *Via International Classification of Diseases, Ninth Revision, codes. **All claims may not have been captured owing to the possibility of patients using providers or pharmacies not in the database. PMTX+: PharMetrics Plus; QoL: quality of life.

We included 3 claims databases in the linkage analysis: the preadjudicated provider- and pharmacy-level (open-source) medical claims (Dx) and prescription claims (Rx) databases and the commercial health plan PharMetrics Plus database of adjudicated medical and pharmacy claims. The databases are characterized in Multimedia Appendix 3. The Dx and Rx databases were merged for the linkage analysis (forming the Dx/Rx database). The quality of the records in the adjudicated, plan-level PharMetrics Plus database is overall higher than the open-source Rx pharmacy and Dx medical claims databases. The latter has the advantage of covering a larger number of patients. An overview of the different types of information captured in the survey and in the individual databases is shown in Figure 2.

To link a completed survey to an entry in any of the claims databases, survey respondents’ data (eg, name, address, zip code, date of birth, sex) were deidentified by means of a multilevel encryption process that was combined with administrative, physical, and technical safeguards to generate unique, encrypted, deidentified tokens that could not be reidentified. The tokens were used in a deterministic matching process to similarly anonymized patients with claims in the IMS Health database. A detailed review of the anonymization and linking methodologies is beyond the scope of this paper, but the methodologies have been used extensively, including in the study cited above [13]. For details, the reader is referred to earlier publications available on the Web [17]. Successful linkage was defined as a match in the claims database to the same anonymous profile in a deterministic process.

### Validation

A total of 4 cohorts were generated for the linkage analysis: cohort 1, all survey participants; cohort 2, survey population successfully linked to the PharMetrics Plus or Dx/Rx claims; cohort 3, survey patients not linkable to claims sources; and cohort 4, patients with MS in PharMetrics Plus. Cohort 4 was made up of all patients with MS in the PharMetrics Plus database aged 18-65 years with ≥1 MS diagnosis and ≥1 month of health plan enrollment between January 1, 2013, and March 31, 2015. For further characterization of linked populations, a subset of linked patients was selected with claims from 2014 or 2015. This was in order to take into account availability of newer therapies and to reduce the discrepancy between the dates of the survey and those of historical claims.

An index date of March 31, 2015 was assigned for all survey patients. For the overall MS population in PharMetrics Plus, the index date was the last month of enrollment between January 1, 2013 and March 31, 2015. For all patients, demographic and clinical characteristics were analyzed and compared for 12 months before the index date.

To validate the representativeness of the cohort identified in the linkage analysis, the degree of concordance between the characteristics of the survey population and those identified in the claims databases was analyzed. Concordance was estimated by calculating the positive predictive values (PPVs; the probability that a claimed characteristic in the survey corresponded to the presence of same characteristic in the linked data). Positive predictive values were calculated as follows: a/(a+b)×100, where a=the number of survey respondents with a specific claim also found in the linked database and b=the total number of survey respondents with the claim. Positive predictive values were calculated for the variables MS diagnosis, current use of disease-modifying therapies (DMTs), prior DMT use, and relapses.

Furthermore, the means and distribution profiles of disease characteristics of all included cohorts were analyzed and compared between the cohorts as follows: cohorts 1 and 4 were compared on demographic characteristics. Cohorts 2 and 4 were compared on clinical characteristics before the index date: use of DMTs, dalfampridine, and corticosteroids (all databases), and comorbidity profiles and Charlson score, use of magnetic resonance imaging of the brain and spine, and relapse rates (PharMetrics Plus only). Cohorts 2 and 3 were compared on all survey results.

### Sample Size

Data on around 302,000 patients with MS were available from the open-source Dx/Rx MS database. The PharMetrics Plus MS database includes data on >100,000 patients in a given year. On the basis of preliminary data and pilot study experience, a 10%-15% linkage rate was expected between the survey and PharMetrics Plus MS cohorts and a linkage rate of >50% to the Dx/Rx cohort. On the basis of these assumptions, a survey sample size of 1000 participants was targeted.

### Statistical Methods

Demographic data were analyzed descriptively. Categorical variables are presented as frequency and percentage (%) of total patients observed in each category. Continuous variables are presented as mean (SD) as well as the median. Statistical significance testing used the chi-square test for categorical variables and the Wilcoxon rank sum test for continuous variables. A *P* value of <.05 was considered statistically significant.

## Results

### Facebook Survey Participants

**Figure 3:**
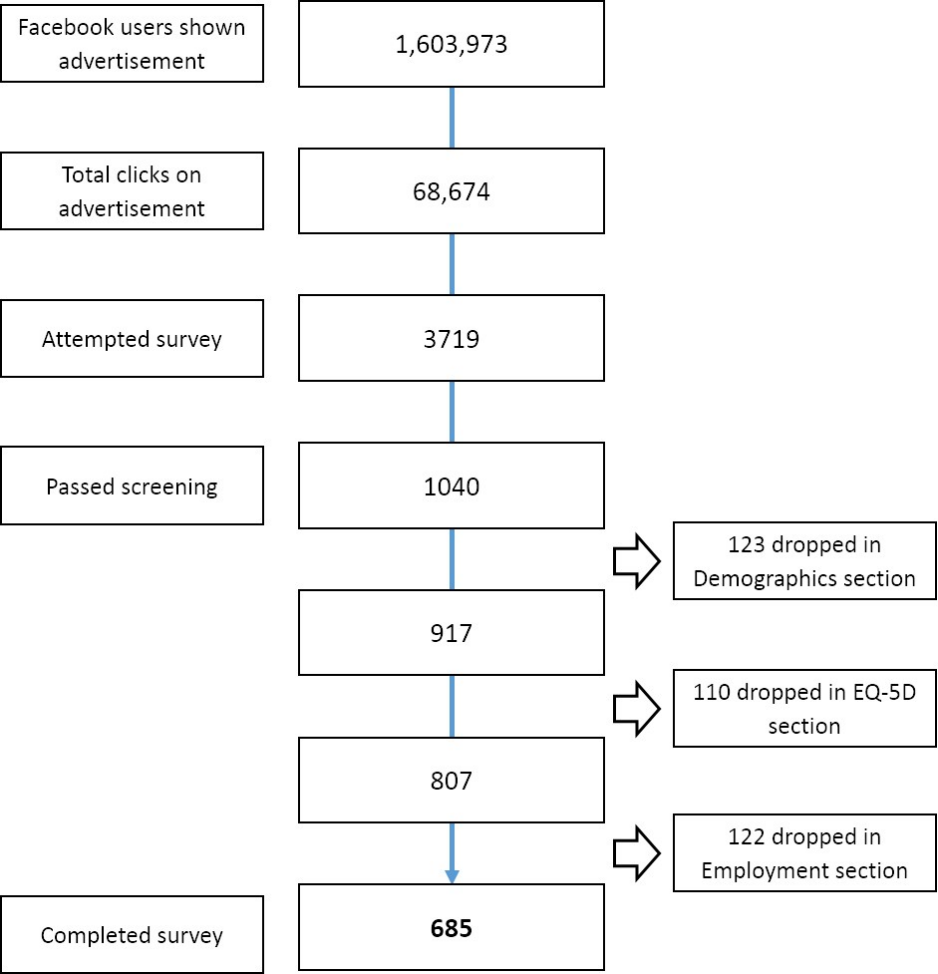
Flow of respondents.

The flow of respondents to the Web-based advertisements and the survey is shown in Figure 3. The Web-based survey was run between July 21, 2015 and September 15, 2015. During this time, the advertisement was placed on 1,063,973 Facebook users’ pages. The advertisements generated a total of 68,674 clicks leading to 3719 attempts at the survey. After filtering out respondents who did not meet the criteria for the survey, 685 respondents completed the survey successfully. The characteristics of 34 respondents were indicative of duplications; thus, 651 unique surveys were included in the linkage analysis (651/1040; 62.60% response rate among eligible respondents who passed screening).

### Data Linkage and Validation

Of the 651 unique patients completing the survey, 453 (69.6%) could be linked with the Dx/Rx database and 73 (11.2%) were linkable to the PharMetrics Plus MS database. A total of 198 survey participants could not be linked; a major reason for this was incorrectly entered data, mostly dates of birth that were missing or incorrect for 67 respondents.

The subset of linked patients with claims from 2014 or 2015 used in further characterization consisted of 440/651 patients (67.6%), 387 of whom (88.0%) were linked to the Dx/Rx database only and 53 (12.0%) to the PharMetrics Plus MS database.

There was a high degree of concordance between the linked patients and the PharMetrics Plus database (Table 1), whereas concordance with Dx/Rx plus PharMetrics Plus was moderate. The PPV for MS diagnosis in the linked patients was 98.1% with PharMetrics Plus (88.0% for all 3 databases), that for current DMT use was 86.5% (51.7%), and that for prior DMT use was 70.0% (47.7%). The PPV for relapses, 34.6%, was lower than that for the other variables.

**Table 1:** Concordance between data from the Web-based survey and the PharMetrics Plus and Dx/Rx + PharMetrics Plus databases.

Variable	PPV^a^ with PharMetrics Plus, %	PPV with Dx/Rx^b^ or PharMetrics Plus, %
Multiple sclerosis diagnosis	98.1	88.0
Any current DMT^c^	86.5	51.7
No current DMT	68.8	86.4
Any prior DMT	70.0	47.7
Relapse in past 12 months	34.6	34.6

^a^PPV: positive predictive value.

^b^Dx/Rx: merged medical claims (Dx) and prescription claims (Rx) databases.

^c^DMT: disease-modifying therapy.

### Generalizability of Survey Data

A comparison of those linkable to the PharMetrics Plus or the Rx/Dx databases in 2014 or 2015 and those not linkable can be found in Table 2. Overall, patients linkable to the open-source databases had slightly greater mean and median age and were more likely to be men than those linkable to PharMetrics Plus. Patients not linkable to the PharMetrics Plus database were more evenly distributed geographically across the United States, a consequence of underrepresentation of this database in the western states (IMS Health internal data). No significant differences were found for educational status, ethnicity, current or prior DMT treatment, or presence of a relapse in the last 12 months between linkable and not linkable individuals.

Because of the complete coverage of health care claims (eg, low likelihood of missing claims compared with open-source databases) captured in the PharMetrics Plus, the additional analysis presented below focuses on the survey patients linked to the PharMetrics Plus MS database.

**Figure 4:**
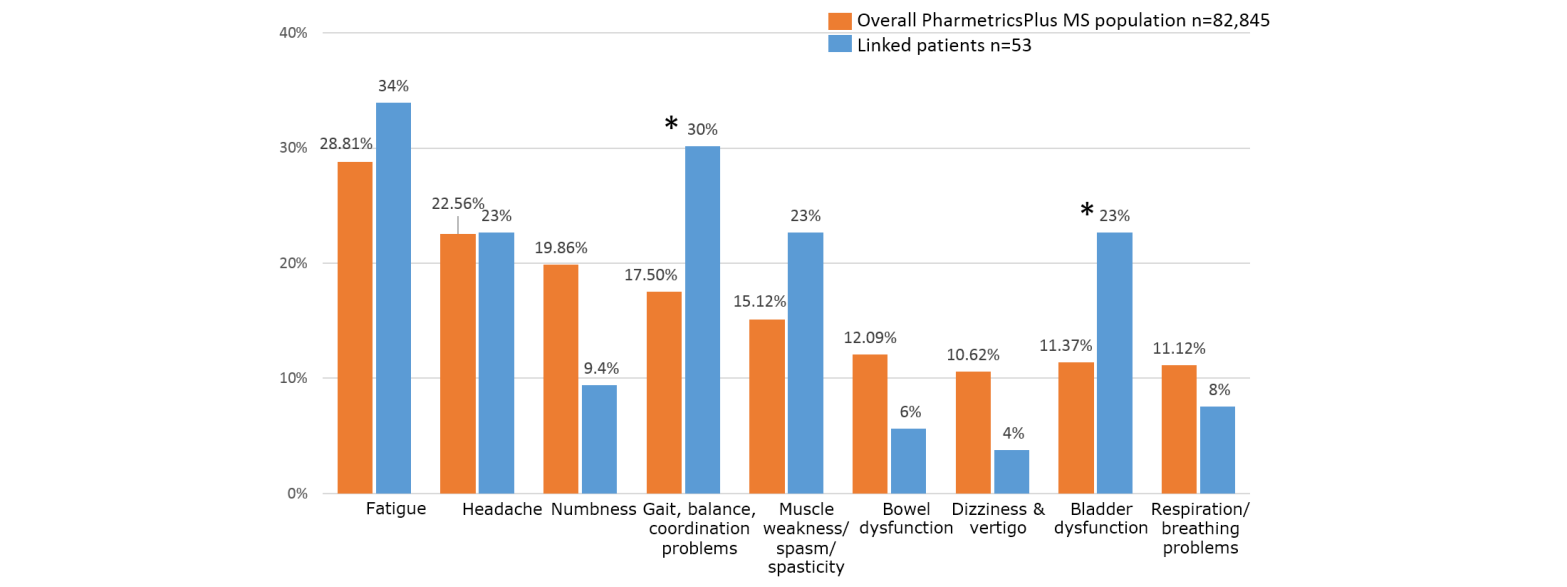
Frequencies of the most common multiple sclerosis (MS) symptoms based on International Classification of Diseases codes on paid claims in the cohort linked to the PharMetrics Plus MS database (blue bars) and the overall PharMetrics Plus MS population (red bars). Only symptoms with prevalence >10% are shown. Asterisk indicates *P*<.05.

Among the most common MS symptoms, the frequencies of a majority of symptoms did not differ significantly between the linked patients and the general PharMetrics Plus MS population (Figure 4). The rate of gait, balance, and coordination problems was higher in the linked population (16/53 or 30% vs 14,500/82,845 or 17.50%; *P*=.015) as was rate of bladder dysfunction (12/53 or 23% vs 9421/82,845 or 11.37%; *P*=.0098). The rate of numbness was lower in the linked population, but this result was not statistically significant (5/53 or 9% vs 16,452/82,845 or 19.86%; *P*=.057). Among comorbidities, only rates of depression differed significantly between the groups, with 21/53 (40%) linked patients reporting depression compared with 22.21% (18,402/82,845) of the overall PharMetrics Plus MS population (*P*=.0023). The proportion of patients with relapses based on a claims-based algorithm in the linked cohort was comparable to that in the overall PharMetrics Plus MS cohort: 11/53 (21%) in the linked cohort versus 15,723/82,845 (18.98%) in the claims database (*P*=.7417).

**Figure 5:**
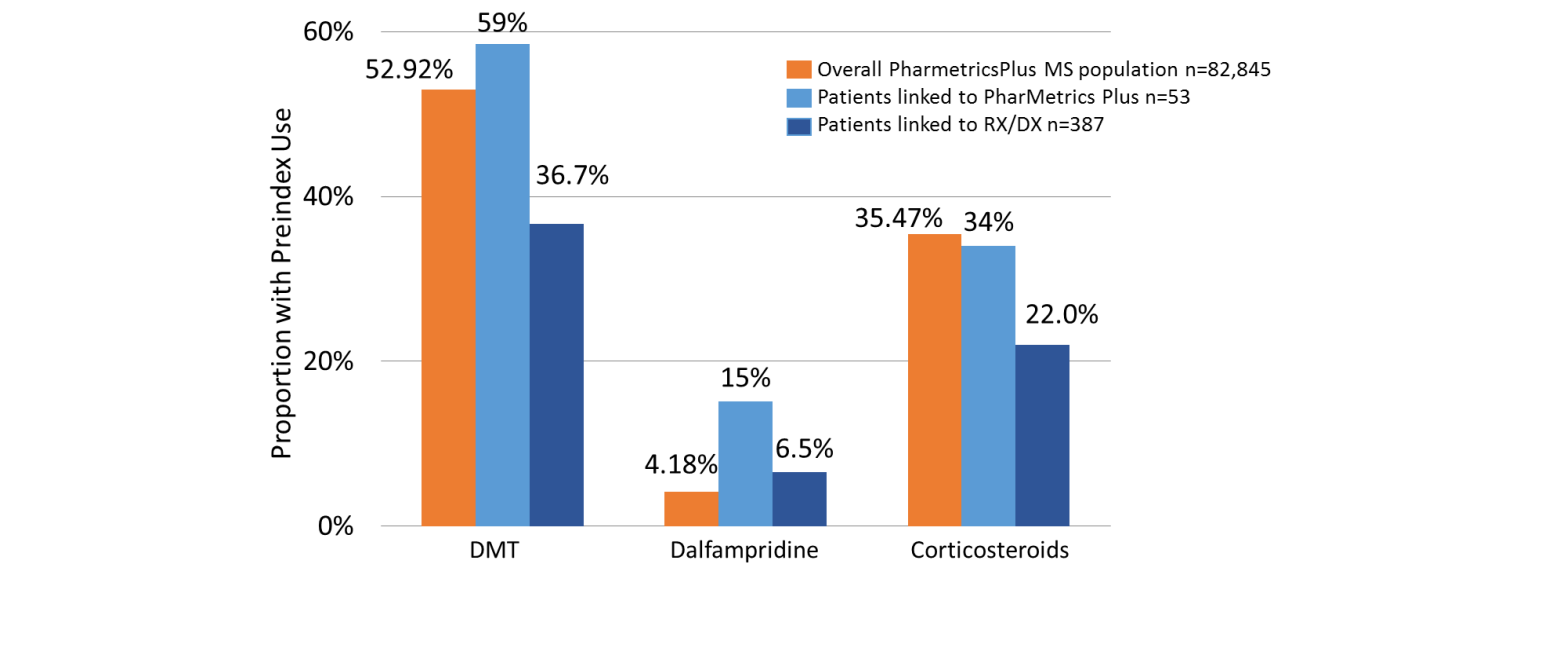
Use of multiple sclerosis (MS)–specific medication in the 1-year index period in the overall PharMetrics Plus MS cohort (red bars) and in the cohorts linked to the PharMetrics Plus MS (light blue bars) and Rx/Dx (dark blue bars) databases. DMT: disease-modifying therapy; Rx/Dx: merged prescription claims (Rx) and medical claims (Dx) database.

Medication use was analyzed for the different populations, displayed graphically in Figure 5. The use of DMTs and corticosteroids was highly similar in the linked cohorts and the overall PharMetrics Plus MS population, whereas more patients in the linked cohort than in the overall population reported dalfampridine use. However, dalfampridine was low in all study populations.

**Table 2:** Demographic and clinical characteristics of the population included in the linkage analysis and the general multiple sclerosis population in the PharMetrics Plus and the Dx/Rx databases, respectively.

Characteristic		Not linkable to PharMetrics Plus or Dx/Rx^a^ (N=211)	Linkable to PharMetrics Plus (N=53)	*P* value	Linkable to Dx/Rx (N=387)	*P* value
Age in years, mean (SD)		46.0 (14.7)	48.9 (8.6)		51.2 (8.8)	
Median age, years		50	49	.79	52.0	.004
Female sex, n (%)		178 (84.4)	46 (87)	.66	318 (82.2)	.50
**Region, n (%)**						
	Northeast	37 (17.5)	18 (34)		75 (19.4)	
	Midwest	51 (24.2)	16 (30)		114 (29.5)	
	South	64 (30.3)	17 (32)		128 (33.1)	
	West	59 (28.0)	2 (4)	<.001	70 (18.1)	.0435
Ethnicity: white, n (%)		186 (88.2)	50 (94)		361 (93.3)	
**Educational status, n (%)**						
	Less than high school	5 (2.4)	1 (2)	.92	7 (1.8)	.25
	Completed high school	43 (20.4)	11 (21)		71 (18.1)	
	Some college	78 (37.0)	17 (32)		141 (36.4)	
	Completed college	65 (30.8)	17 (32)		106 (27.4)	
	Graduate school	20 (9.5)	7 (13)		62 (16.0)	
**MS^b^** **subtype, n (%)**						
	RRMS^c^	173 (82.0)	46 (87)	.22	317 (81.9)	>.99
	PPMS^d^	14 (6.6)	5 (9)		25 (6.5)	
	SPMS^e^	24 (11.4)	2 (4)		45 (11.6)	
**Time since diagnosis, n (%)**						
	>1 year	47 (22.3)	9 (17)	.04	119 (30.7)	.06
	>5 years	79 (37.4)	11 (21)		118 (30.5)	
	>10 years	85 (40.3)	33 (62)		150 (38.3)	
**DMT^f^** **treatment, n (%)**						
	No DMT	45 (21.3)	16 (30)		72 (18.6)	
	Copaxone and interferons	84 (39.8)	12 (23)		140 (36.2)	
	Oral DMT^g^	57 (27.0)	17 (32)		130 (33.6)	
	Infused DMT^h^	21 (10.0)	8 (15)		41 (10.6)	
**Duration of current treatment, n (%)**						
	1-5 years	145 (68.7)	41 (77)		295 (76.2)	
	6-10 years	40 (19.0)	7 (13)		50 (12.9)	
	11-15 years	12 (5.7)	2 (4)		24 (6.2)	
	16-20 years	10 (4.7)	1 (2)		14 (3.6)	
	>20 years	4 (1.9)	2 (4)		4 (1.0)	
**Prior DMT treatment, n (%)**						
	No DMT	67 (31.8)	16 (30)	.83	99 (25.6)	.11
	Copaxone and interferons	148 (70.1)	43 (60)		296 (76.5)	
	Oral DMT	21 (10.0)	11 (21)		32 (8.3)	
	Infused DMT	22 (10.4)	5 (9)		34 (8.8)	
≥1 Relapse, n (%)		117 (55.5)	26 (49)		230 (59.4)	

^a^Dx/Rx: merged medical claims (Dx) and prescription claims (Rx) database.

^b^MS: multiple sclerosis.

^c^RRMS: relapsing-remitting multiple sclerosis.

^d^PPMS: primary progressive multiple sclerosis.

^e^SPMS: secondary progressive multiple sclerosis.

^f^DMT: disease-modifying therapy.

^g^Oral DMTs include Gilenya, Tecfidera, and Aubagio.

^h^Infused DMTs include Tysabri and Lemtrada.

## Discussion

### Principal Findings

The aim of this study was to explore the feasibility of creating a dataset that contains both patient outcomes data (from a Web-based survey of US patients with MS recruited on an open Internet platform) and health care utilization information from pharmacy and medical claims databases. The initial results presented here indicate that this aim was fulfilled and that the survey population is broadly representative of the general MS patient population in the United States.

People with MS are highly active on social media [18,19]. We have previously shown that the demographics of cohorts of patients with MS identified by their activities on social media correspond well with cohorts identified in other sources, indicating that social media data analysis can be usefully applied to outcomes research [12]. Facebook, forums, and blogs have been used previously to recruit participants into surveys of health outcomes and lifestyle interventions in MS [20-22]. Our work differs in a number of important aspects from previous reports, however. First, patients were recruited through unbranded advertisements placed on Facebook pages, not by active, personal invitations sent to specific target groups. Although this approach led to a markedly lower rate of successfully completed surveys than that reported with patients invited directly, the absence of active targeting of participants can be expected to reduce the scope for bias and generate a more representative population.

The same differences apply to a very recent report that described successful linking of data from invited patients on a dedicated online patient community (PatientsLikeMe) with administrative claims data [13]. In contrast to our approach, targeted invitations were sent to eligible patients identified on the social network by email and private messages. Both approaches have their merits, but the population from the online patient community may well have been less diverse than patients who can be reached by untargeted advertisements on open Internet platforms such as Facebook.

The growing interest in linking health care–related social media content to information obtained by traditional means, for example, claims databases, reflects the realization that such linked data may provide a rapid, cost-effective, and credible method to capture patient outcomes, behavioral data, and health care claims. Our use of a standardized survey allowed us to overcome a common limitation of data from open Internet platforms: a lack of structured clinical, socioeconomic, and demographic data necessary for observational research [23]. The survey allowed us to obtain structured, disease-specific information on disease duration, medications, disabilities, and impact of the disease on quality of life as well as work productivity. The linkage to claims databases generated a dataset that combines patient-reported MS-related information and standardized data driven by the claims classification system. The linked database thus includes a wealth of information that is typically only available in separate databases. This could enable deeper and more rounded insights into burden of illness and other outcomes beyond what is possible with conventional database approaches. For example, complementing claims databases with patient-derived information on MS disease type would enable analyses of the impact of different types of MS on productivity or disability. Such information cannot be obtained from either data source in isolation. The potential to derive outcomes data was not assessed in this study but is currently being explored in future analysis of the database.

In our analysis, the concordance of the data, measured as PPV, between survey responses and both the claims databases used was high for diagnosis and current and prior DMT but lower for relapses. In the open-source database, relapse was based on a claims-based algorithm [24,25]. The survey was based on patient recall, which is typically less exact than data entered into claims databases.

The size of the cohort and the percentage of survey respondents linkable to the PharMetrics Plus database were relatively modest. There are several reasons for this, none of which invalidates the approach. The odds of successful linkage depend on the size and population coverage of the databases selected. Although it has a lower likelihood of missing claims, PharMetrics Plus is an order of magnitude smaller than the open-source databases and underrepresents patients from the western United States, which reduced the potential to link survey respondents from this region. These are weaknesses specific to the specific database, not to the method.

When expanding the linkage methodology to include databases such as electronic medical records [26], it is important to take the privacy aspect into account. It has been strongly argued that important privacy concerns must be interpreted alongside the social good that can come from this kind of health research [27-30]. In this study, the patient survey required active opt-in from the participants and no data were obtained from participants’ Facebook accounts.

### Limitations

There are weaknesses in the analysis. The sample size of 53 patients linked to the PharMetrics Plus database is too small to read much into the data, and the main value of this particular dataset is in demonstrating the feasibility of the method. The high linkage rates but low concordance with the Dx/Rx databases are noted, attributable to the open-source nature of the databases. As social media content is user driven, there is no independent verification of the correctness of the data, although the concordance analysis indicates that the social media information reflected patients’ actual situations. This limitation applies to all social media analyses [31]. There is a wide range of trust in Web-based information [19], and differences in attitudes toward social media among patients with MS may produce a certain bias in the survey population toward those more willing to use and trust social media. All data are for US populations and the generalizability of the methods has yet to be established. There is a possibility of selection bias as participants were recruited by Web-based advertisements. From the survey results, the Facebook-recruited survey participants may have been somewhat more severely affected by their illness than the overall population. More symptomatic patients may be more motivated to complete a Web-based survey. Such bias toward more severely affected patients would also affect an analysis of MS-related costs. It should also be underlined that the survey relied on patient recall, which is less than 100% reliable [32]. The date for an event recorded by a patient in the survey may not correspond to the time point for the same event in the PharMetrics Plus database. However, given the large number of participants and the relatively high degree of linkage, these two risks do not seem to have invalidated the collected data.

With these limitations in mind, this study shows that the combination of advertisements on open Internet platforms and Web-based surveys may enable rapid gathering of real-life data on a large US population of representative patients with MS. The applicability of the approach to diseases other than MS would need independent verification.

## Appendix

Multimedia Appendix 1 Survey questionnaire. The EQ-5D-3L and EQ-VAS instruments are proprietary and cannot be displayed in publicly available materials.

Multimedia Appendix 2 Disclaimer presented to survey participants explaining confidentiality and handling of the data.

Multimedia Appendix 3 Description of the databases used in the analysis.

## Abbreviations

DMT: disease-modifying therapy
HIPAA: Health Insurance Portability and Accountability Act
https: hypertext transfer protocol, secure
MS: multiple sclerosis
PPV: positive predictive value
STROBE: Strengthening the Reporting of Observational Studies in Epidemiology
