# Supplementary material for: Linked Patient-Reported Outcomes Data From Patients With Multiple Sclerosis Recruited on an Open Internet Platform to Health Care Claims Databases Identifies a Representative Population for Real-Life Data Analysis in Multiple Sclerosis
Source: J Med Internet Res. 2016 Sep 22;18(9):e249. doi: 10.2196/jmir.5805 (PMC5054235; doi:10.2196/jmir.5805)
Supplement: Multimedia Appendix 1 [file jmir_v18i9e249_app1.pdf]

Survey questionnaire

1.1 Demographics and disease characteristics

- **Please enter your details**
    I. First Name
    II. Last name
    III. Address
    IV. City
    V. ZIP code

- **Please select your state**
  (Drop down for 50 US States)

- **Enter your Date of birth**
  (Calendar year from 1900 → 2015)

- **What is your gender?**
  (Male/Female)

- **What is your ethnicity?**
  (American Indian or Alaska Native, Asian, Black or African American, Native Hawaiian or Other Pacific Islander, White)

- **Educational status**
  (Less than high school/Completed high school/Some college/Completed college/Graduate school)

- **For how many years have you had Multiple Sclerosis (time since first symptom)?**
  (range from 1 to 20 and >20)

- **When was your Multiple Sclerosis diagnosed (year)?**
  (Year range from1950 to 2015)

- **What Disease Modifying Therapy (DMT) are you currently on?**
    - Aubagio (Teriflunomide)
    - Avonex Pen (interferon beta-1a)
    - Betaseron (interferon beta-1b)

- Extavia(interferon beta-1b)
- Gilenya (Fingolimod)
- Glatiramer Acetate (Copaxone)
- Lemtrada (Alemtuzumab)
- Plegridy (Interferons)
- Rebidose with pen (interferon beta -1a)
- Rebif without pen (interferon beta -1a)
- Tecfidera (Dimethyl Fumarate)
- Tysabri (Natalizumab)
- Not on DMT

- **How many years have you been on this therapy?**
  (range from 1 to 20 and >20)
- **What Disease Modifying Therapy (DMT) were you on prior to this treatment?**

  Aubagio (Teriflunomide)
  Avonex (interferon beta-1a)
  Betaseron (interferon beta-1b)
  Extavia(interferon beta-1b)
  Gilenya (Fingolimod)
  Glatiramer Acetate (Copaxone)
  Lemtrada (Alemtuzumab)
  Plegridy (Interferons)
  Rebidose with pen (interferon beta -1a)
  Rebif without pen (interferon beta -1a)
  Tecfidera (Dimethyl Fumarate)
  Tysabri (Natalizumab)
  Not on DMT

- **How many relapses (deterioration in health condition after period of improvement) have you had in last 12 months?**
  (range from 0 to 20 and >20)
  If respondents had at least one relapse, addition 2 questions will be displayed for respondent to answer
- **Were you hospitalized during one of these relapses?**
  - Yes: Emergency Room Visit
  - Yes: In-patient stay
  - Yes: Both emergency room visit and in-patient stay
  - No
- **Were you given steroids during one of these relapses? Oral, IV, both or none?**
  (Oral, IV, Both, No steroids were taken)

1.2 Disease severity

Please read the choices listed below and choose the one that best describes your own situation. This scale focuses mainly on how well you walk. You might not find a description that reflects your condition exactly, but please mark the one category that describes your situation the closest.

☐ **Normal:** I may have some mild symptoms, mostly sensory due to MS but they do not limit my activity. If I do have an attack, I return to normal when the attack has passed.

☐ **Mild Disability:** I have some noticeable symptoms from my MS but they are minor and have only a small effect on my lifestyle.

☐ **Moderate Disability:** I don't have any limitations in my walking ability. However, I do have significant problems due to MS that limit daily activities in other ways.

☐ **Gait Disability:** MS does interfere with my activities, especially my walking. I can work a full day, but athletic or physically demanding activities are more difficult than they used to be. I usually don't need a cane or other assistance to walk, but I might need some assistance during an attack.

☐ **Early Cane:** I use a cane or a single crutch or some other form of support (such as touching a wall or leaning on someone's arm) for walking all the time or part of the time, especially when walking outside. I think I can walk 25 feet in 20 seconds without a cane or crutch. I always need some assistance (cane or crutch) if I want to walk as far as 3 blocks.

☐ **Late Cane:** To be able to walk 25 feet, I have to have a cane, crutch or someone to hold onto. I can get around the house or other buildings by holding onto furniture or touching the walls for support. I may use a scooter or wheelchair if I want to go greater distances.

☐ **Bilateral Support:** To be able to walk as far as 25 feet I must have 2 canes or crutches or a walker. I may use a scooter or wheelchair for longer distances.

☐ **Wheelchair / Scooter:** My main form of mobility is a wheelchair. I may be able to stand and/or take one or two steps, but I can't walk 25 feet, even with crutches or a walker.

☐ **Bedridden:** Unable to sit in a wheelchair for more than one hour.

Loading EQ-5D questionnaire.....The next part of the questionnaire is currently loading, please wait as this may take a few minutes. Please do not press the back button on your browser while you wait. Thank you for your patience

1.3 Health related QoL via EQ5D-3L

This questionnaire is under copyright.

Health related QoL via EQ-VAS

This questionnaire is under copyright.

1.4 Employment status and Productivity

Work Productivity and Activity Impairment Questionnaire: Multiple Sclerosis V2.1 (WPAI:MS)

1. **Are you currently employed (working for pay)?**
(Yes/ No)
If NO, tick "NO" and skip to question 6.

The next questions are about the **past seven days**, not including today.

2. During the past seven days, how many hours did you miss from work because of problems associated with your multiple sclerosis? Include hours you missed on sick days, times you went in late, left early, etc., because of problems associated with multiple sclerosis. Do not include time you missed to participate in this study. (Please enter value in hours)
_____ HOURS

3. During the past seven days, how many hours did you miss from work because of any other reason, such as vacation, public holidays? (Please enter value in hours)

_____ HOURS

4. During the past seven days, how many hours did you actually work? (Please enter value in hours)

_____ HOURS

(If "0", skip to question 6.)

5.* During the past seven days, how much did your multiple sclerosis affect your productivity while you were working? *Think about days you were limited in the amount or kind of work you could do, days you accomplished less than you would like, or days you could not do your work as carefully as usual. If multiple sclerosis affected your work only a little, choose a low number. Choose a high number if multiple sclerosis affected your work a great deal.

(0 indicates - Multiple sclerosis had no effect on my work, 10 indicates - Multiple sclerosis completely prevented me from working)

Multiple sclerosis had no effect on my work | 0 1 2 3 4 5 6 7 8 9 10 | Multiple sclerosis completely prevented me from working

6. During the past seven days, how much did your multiple sclerosis affect your ability to do your usual daily activities, other than work at a job? *By usual activities, we mean the normal activities you do, such as work around the house, shopping, childcare, exercising, studying, etc. Think about times you were limited in the amount or kind of activities you could do and times you accomplished less than you would like. If multiple sclerosis affected your activities only a little, choose a low number. Choose a high number if multiple sclerosis affected your activities a great deal.*

(0 indicates - Multiple sclerosis had no effect on my daily activities,

10 indicates - Multiple sclerosis completely prevented me from doing my daily activities)

Multiple sclerosis had no effect on my daily activities | 0 1 2 3 4 5 6 7 8 9 10 | Multiple sclerosis completely prevented me from doing my daily activities

Thank you
