# Supplementary material for: Linked Patient-Reported Outcomes Data From Patients With Multiple Sclerosis Recruited on an Open Internet Platform to Health Care Claims Databases Identifies a Representative Population for Real-Life Data Analysis in Multiple Sclerosis
Source: J Med Internet Res. 2016 Sep 22;18(9):e249. doi: 10.2196/jmir.5805 (PMC5054235; doi:10.2196/jmir.5805)
Supplement: Multimedia Appendix 2 [file jmir_v18i9e249_app2.pdf]

# Disclaimer

## Burden of Illness Research Study in Multiple Sclerosis

We welcome you to complete this approximately **10 minute survey** to help us better understand and quantify the impact of disease in patients with Multiple Sclerosis. Importantly your answers will be anonymous and included into a larger patient-centric research study, which is sponsored by Novartis Pharma AG, Switzerland. For the purposes of this study Novartis will only gain access to aggregated information and will be unable to see any individual responses.

Upon answering all the questions, you will receive an Amazon gift voucher of $10, which will be mailed to your address within 4 weeks of completing the survey. The confidentiality of the information received as part of this survey will be maintained at all times.

By answering the questions below, you agree to authorize IMS Health, which includes its representatives and agents to use and disclose any or all of the information provided by me, including, for the following purposes:

(1) to communicate with me and facilitate payment for my participation in the survey

(2) to conduct a survey on my experience with Multiple Sclerosis;

(3) in aggregate form, to compile reports or other communications related to the survey and its results;

I understand that the information I provide to IMS Health in connection with this survey may include, without limitation, information about my medical history, and about the medical services and treatments I have received.

For clarity, I also understand that my name and contact information will not appear in any report or communication except communications between me and IMS to facilitate payment for my participation in this survey. I understand that recipients of my information may not be governed by any federal privacy laws.

IMS HEALTH will treat the information you provide to us including during the survey ("your data") in a confidential manner. Your contact details will solely be used to facilitate payment for your participation in this survey, and will not be provided to any third parties. In addition, IMS HEALTH has taken the appropriate administrative and technical and organizational measures for the protection of confidential data.

**Revocation.**

**Right to Revoke:** You may revoke this authorization at any time by giving written notice of revocation to the IMS Health Contact Office listed below. Revocation of this authorization will *not* affect any action we took in reliance on this authorization before we received your written notice of revocation. For clarity, we will not be able to remove your information from aggregate information compiled from the information of survey participants prior to receipt of your written notice of revocation to Support-NexxusSocial@imshealth.com.

*Note:*

*The screener questions in the first section of the survey will identify the suitable respondents to take part in the survey. Only the eligible respondents are qualified to complete the survey and hence entitled to receive a Amazon gift voucher.*

*You must complete the survey fully to become entitled for the gift voucher.*

*Once the survey is started, it cannot be closed until all questions are completed or re-done at another time. If the survey is closed before the end then you will not be eligible for the gift voucher.*

*Please ensure that you provide accurate details.*

*Thank you for your participation in this exciting research initiative.*
