# Supplementary material for: Linked Patient-Reported Outcomes Data From Patients With Multiple Sclerosis Recruited on an Open Internet Platform to Health Care Claims Databases Identifies a Representative Population for Real-Life Data Analysis in Multiple Sclerosis
Source: J Med Internet Res. 2016 Sep 22;18(9):e249. doi: 10.2196/jmir.5805 (PMC5054235; doi:10.2196/jmir.5805)
Supplement: Multimedia Appendix 3 [file jmir_v18i9e249_app3.pdf]

Database	Medical Claims (Dx)	Pharmacy Prescription Claims (Rx)	PharMetrics Plus medical and pharmacy claims
Data sources and size	Collects ~one billion professional fee medical claims per year, representing over 870,000 practitioners per month<br>Data obtained through agreements with electronic claims re-processors	> 1.6 billion retail, mail service and specialty pharmacy prescription claims<br>Represents dispensed prescriptions for ~55% of all U.S. pharmacy transactions	Adjudicated medical and pharmacy claims for >87 million health plan members across the United States<br>Representative of the US commercially insured population with broad geographic coverage<br>Specifically includes integrated claims data
Data gathered	Diagnoses, procedures and office-administered drugs submitted on CMS-1500 professional fee claims, for insured patients across all types of payers	Claims include those reimbursed by cash, Medicare, Medicaid and other third-party transactions	Longitudinal data<br>~22 million patients with ≥4 years of continuous enrolment in their health plans<br>Includes ~187,000 patients with MS, >106,000 of whom have a linkable identity. Believed to be representative of the population of

			patients with MS in the United States (IMS Health internal analysis)
Time covered	Records available from September 1999 through July 2015<br>~95% of claims available for analyses within 3 weeks of the service date	Records available from April 2001 through July 2015<br>~95% of claims are available for analyses within 12 days of being dispensed	Data available from Jan 1, 2006 to Mar 31, 2015

Description of the databases included in the linkage analysis. The Dx and Rx databases were merged for the linkage analysis
